# Biochemical and Structural Characterization of Enolase from *Chloroflexus aurantiacus:* Evidence for a Thermophilic Origin

**DOI:** 10.3389/fbioe.2015.00074

**Published:** 2015-06-01

**Authors:** Oleg A. Zadvornyy, Eric S. Boyd, Matthew C. Posewitz, Nikolay A. Zorin, John W. Peters

**Affiliations:** ^1^Department of Chemistry and Biochemistry, Montana State University, Bozeman, MT, USA; ^2^Institute of Basic Biological Problems, Russian Academy of Sciences, Pushchino, Russia; ^3^Department of Microbiology and Immunology, Montana State University, Bozeman, MT, USA; ^4^Department of Chemistry and Geochemistry, Colorado School of Mines, Golden, CO, USA

**Keywords:** enolase, thermal stability, origin, evolution, hydropathy, green sulfur bacteria

## Abstract

Enolase catalyzes the conversion of 2-phosphoglycerate to phosphoenolpyruvate during both glycolysis and gluconeogenesis, and is required by all three domains of life. Here, we report the purification and biochemical and structural characterization of enolase from *Chloroflexus aurantiacus*, a thermophilic anoxygenic phototroph affiliated with the green non-sulfur bacteria. The protein was purified as a homodimer with a subunit molecular weight of 46 kDa. The temperature optimum for enolase catalysis was 80°C, close to the measured thermal stability of the protein which was determined to be 75°C, while the pH optimum for enzyme activity was 6.5. The specific activities of purified enolase determined at 25 and 80°C were 147 and 300 U mg^−1^ of protein, respectively. *K*_m_ values for the 2-phosphoglycerate/phosphoenolpyruvate reaction determined at 25 and 80°C were 0.16 and 0.03 mM, respectively. The *K*_m_ values for Mg^2+^ binding at these temperatures were 2.5 and 1.9 mM, respectively. When compared to enolase from mesophiles, the biochemical and structural properties of enolase from *C. aurantiacus* are consistent with this being thermally adapted. These data are consistent with the results of our phylogenetic analysis of enolase, which reveal that enolase has a thermophilic origin.

## Introduction

Enolase (2-phospho-d-glycerate hydrolyase, EC 4.2.1.11) catalyzes the conversion of 2-phosphoglycerate (2-PGA) to phosphoenolpyruvate (PEP) during both glycolysis and gluconeogenesis in all three domains of life (Ballou and Wold, 1957; Wold, 1971). Enolase is a metalloenzyme activated by cations of bivalent metals (Brewer, 1981), such as magnesium (Mg^2+^). In bacteria, enolases are highly conserved enzymes and commonly exist as homodimers with molecular weights in the range of 80–100 kDa; the mass of a single subunit ranges from 40 to 50 kDa. Intriguingly, purified enolase from the thermophilic bacteria *Thermotoga maritima* (Schurig et al., 1995) and *Thermus aquaticus* (Stellwag et al., 1973) have been reported to be octamers. However, enolase from the anaerobic, hyperthermophilic archaeon *Pyrococcus furiosus* was reported as a homodimer (Peak et al., 1994).

Crystal structures of enolase from a variety of taxonomic sources have been examined; however, the majority of studies have been focused on the structure of the homodimeric enolase from *Saccharomyces cerevisiae* (Chin et al., 1981; Lebioda et al., 1991; Wedekind et al., 1995; Brewer et al., 1998; Sims et al., 2006; Schreier and Hocker, 2010). In the *S. cerevisiae* enolase dimeric structure, each monomer consists of a carboxyl terminal catalytic site (Lebioda and Stec, 1988; Lebioda et al., 1989; Stec and Lebioda, 1990; Lebioda and Stec, 1991; Zhang et al., 1994), which is highly conserved in enolases from different microorganisms. In addition, upon binding of the substrate to the active site, several conformations of the loop regions near the active site have been observed in the structures of this enzyme. When co-crystallized with Mg^2+^ and 2-PGA or PEP, the *S. cerevisiae* enolase structure adopts a completely “closed” state. In the closed state, the flexible active site loops L1 (residues 36–43) from the lid domain and the L2 (residues 153–169) and L3 (residues 251–277) loops from the barrel domain are all in a closed conformation (Figure 3) (Larsen et al., 1996; Zhang et al., 1997; Sims et al., 2006). In contrast, in the apo state, the L1 motif is far removed from the active site and the L2 and L3 loops are in the “open” conformation (Lebioda and Stec, 1991). In addition to the dimeric structure described above, enolase has been shown to form asymmetric dimers in which the subunits adopt two different conformations (Sims et al., 2006; Schulz et al., 2011).

The catalytic mechanism of enolase has been studied in a number of phylogenetically distinct organisms, including representatives from Archaea, Bacteria, and Eukarya (Wold and Ballou, 1957b; Brewer, 1981; Reed et al., 1996; Zhang et al., 1997). From such studies, it is clear that all members of this superfamily share a common initial reaction step: the abstraction of the *R*-proton of a carboxylate substrate by a general base (Babbitt et al., 1996; Gerlt et al., 2005), represented by lysine 345 in *S. cerevisiae* enolase (Poyner et al., 1996). The resulting enolic intermediate is stabilized by a magnesium ion [Mg^2+^(I)], in the conserved active site that interacts with the intermediate carboxylate group. Enolase is unique in that it is the only member of the enolase superfamily in which a reaction intermediate is coordinated by a second catalytic magnesium ion [Mg^2+^(II)]. Mg^2+^(II) interacts with one carboxylate oxygen and a phosphate group oxygen of the substrate 2-PGA. In *S. cerevisiae* enolase, serine 39 in the L1 motif of the lid domain is the only residue that directly interacts with Mg^2+^(II), while two water molecules positioned by aspartate 321 complete the coordination sphere of Mg^2+^(II) (Zhang et al., 1994; Larsen et al., 1996). Both magnesium ions [e.g., Mg^2+^(I) and Mg^2+^(II)] are thought to participate in the crucial first step of the enolase reaction, the ionization of 2-PGA to give the negatively charged enolic intermediate and the stabilization thereof.

In the second step of the enolase reaction, the general acid glutamate 211 facilitates the dissociation of the hydroxide to form PEP (Larsen et al., 1996; Poyner et al., 1996; Reed et al., 1996). Enolase mutants in which serine at the position 39 in the L1 loop is substituted for asparagine retain basal catalytic enolase activity with coordination of Mg^2+^(I) and 2-PGA in an “open” active site that does not require the Mg^2+^(II) coordination residues (Schreier and Hocker, 2010). Interestingly, a structure of enolase from the anaerobic protozoan *Entamoeba histolyca* contains 2-PGA in the active site and exists in the open conformation; the Mg^2+^(II) ion is absent from the active site (Schulz et al., 2011).

The widespread taxonomic distribution of enolase in Bacteria and Archaea (Tracy and Hedges, 2000), coupled with its fundamental role in glycolysis and gluconeogenesis (Wold, 1971; Fothergill-Gilmore and Michels, 1993; Ronimus and Morgan, 2003), strongly suggests that enolase was present in the Last Universal Common Ancestor (LUCA) of Bacteria and Archaea. Evidence derived from the characteristics of deeply branching taxa on the universal tree of life suggests that LUCA may have been a thermophile (Pace, 1991; Ronimus and Morgan, 2003; Lineweaver and Schwartzman, 2003). Proteins isolated from thermophilic microorganisms exhibit properties relative to their mesophilic counterparts that allow them to function in these extreme environments (Miller, 2003).

In the present study, we purified enolase-1 from *Chloroflexus aurantiacus* (EnoCa), a thermophilic green non-sulfur bacterium that grows photosynthetically under anaerobic conditions. Members of the green sulfur bacteria are thought to have emerged early in the evolution of photosynthetic metabolisms, whereby green sulfur bacteria gave rise to gram positive *Heliobacteriales* capable of photosynthesis, followed by the emergence of photosynthesis in cyanobacteria (Gupta et al., 1999; Xiong et al., 2000). Detailed biochemical and structural analysis of EnoCa reveal features that are consistent with adaptation to high temperature. These results, in the context of our phylogenetic work indicating enolase has a thermophilic origin, confirm adaptation of this enzyme to high temperature and suggest that EnoCa emerged from a thermophilic ancestor. Comparison of biochemical and structural features of EnoCa with enolase from phylogenetically diverse microorganisms reveal a number of common features that are likely to confer thermostability to members of this enzyme superfamily.

## Materials and Methods

### Growth conditions of *C. aurantiacus*

*Chloroflexus aurantiacus* strain J.10.fl. (courtesy of Dr. Mikhail F. Yanyushin) was grown in 1 L screw-capped bottles illuminated by two pairs of 100 W incandescent lamps at 56°C in a modified Castenholz Medium (Castenholz, 1969; Yanyushin, 1988) (Tables S1 and S2 in Supplementary Material), or in 100 L fermenters stirred at 200 rpm and bubbled with nitrogen gas passed through a 0.2 μm filter (Fisher Scientific, Ireland). Fermenters were illuminated by five 150 W incandescent lamps.

### Purification of EnoCa

Cultures of *C. aurantiacus* were harvested in mid-exponential growth phase by centrifugation (6,000 × *g*, 25 min). Cell pellets (100 g) were washed twice with Tris-HCl buffer (50 mM, pH 8.0). Following washing, the cell pellet was re-suspended in Tris-HCl buffer (50 mM, pH 8.0) and sonicated using a Branson Sonifier 450 (VWR Scientific, USA) at 40% power for 5 min at 4°C. This process was repeated two additional times. Unbroken cells and cell fragments were pelleted by centrifugation (14,000 × *g*, 40 min, 4°C). Following centrifugation, the cell free extract was diluted 10-fold with Tris-HCl buffer (5 mM, pH 8.0), and ~570 mg were applied to Q-sepharose column (GE Healthcare, Sweden) equilibrated with Tris-HCl buffer (50 mM, pH 8.0). A linear gradient of 0.05–1.00M NaCl in Tris-HCl buffer (50 mM, pH 8.0) was applied to the column at a flow rate of 3.5 ml min^−1^. Enolase fractions with activity eluted at ~0.5M NaCl. These fractions were combined and concentrated using a Molecular Stirred cell (Spectrum Laboratories, Inc., USA). Concentrated protein (~250 mg) was loaded onto a Sephacryl S-300 (Pharmacia, Sweden) gel filtration column (2.5 × 100 cm) at a flow rate 1 ml min^−1^. Fractions that exhibited enolase activity were combined and subjected to further purification using a hydrophobic Octyl Sepharose column (GE Healthcare, Sweden). The column was equilibrated with 0.8M NaCl in Tris-HCl buffer (50 mM, pH 8.0). A linear gradient of 0.8–0.0M NaCl was applied to the column with a flow rate 2.5 ml min^−1^. The active enolase fractions were combined and desalted using a Sephadex G-25 (GE Healthcare, Sweden). The purity of protein sample was confirmed by SDS-PAGE. Purified enolase was stored in liquid nitrogen until further biochemical and structural characterization.

### EnoCa protein concentration and kinetic assays

The concentration of protein in these samples was determined using the Bradford Assay (Bradford, 1976) with bovine serum albumin as a standard. The activity of purified EnoCa was determined by monitoring the conversion of 2-PGA to PEP. PEP absorbs at 240 nm and was quantified over time in a temperature-controlled assay using a Cary50-Bio-UV-Visible spectrophotometer. The assay contained 1.5 mM PGA, 5 mM MgCl_2_ in Bis-Tris propane (50 mM, pH 6.5), and enolase (12 μg), unless otherwise stated. The change in PEP concentration was determined using an absorption coefficient (ε_240-25t_) = 1.7 mM^−1^ cm^−1^ at 25°C and (ε_240-80t_) = 1.2 at mM^−1^ cm^−1^ at 80°C. The absorption coefficient of PEP varies with pH, concentration of Mg^2+^, and temperature. Corrected molar absorptivity for PEP was used in experiments where pH, Mg^2+^ concentration, and temperature were varied (Wold and Ballou, 1957a,b). One unit (U) of the enzyme activity was defined as the amount of enolase that converts 1 μmol of 2-PGA into PEP in 1 min at 80°C, unless otherwise stated. Michaelis–Menten kinetic parameters were determined from curves generated by plotting the concentration of substrate p as a function of reaction velocity. The standard reaction mixture contained 1.5 mM 2-PGA, 5 mM MgCl_2_ in 50 mM Bis-Tris propane (pH 6.5), and enolase sample (12 μg). The 2-PGA concentrations varied from 0.04 to 12 mM, while Mg^2+^ concentrations ranged from 0.05 to 20 mM. The reaction was initiated by the addition of 12 μg of enzyme. To determine the Mg^2+^ kinetic parameters, the enzyme was subjected to an additional round of purification using a PD-10 (Sephadex™ G-25, GE Healthcare, Sweden) desalting column equilibrated with Bis-Tris propane buffer (50 mM, pH 6.5) free of Mg^2+^. To investigate the effect of the Tris-HCl (50mM, pH 8.0), HEPES (50 mM, pH 8.0), or Bis-Tris propane (50 mM, pH 8.0) buffer on the enolase activity, the protein was exchanged on Sephadex G-25 column equilibrated with the corresponding buffer.

### Thermal stability and temperature optimum of enzyme

To investigate the thermal stability of the EnoCa, protein samples were heated for 5 min at the specified temperature (25–90°C), and then placed immediately on ice before being added to the reaction mixture. The activity of heat-treated enzymes was determined using the methods described above at a temperature of 25°C. The optimum temperature for the activity of enolase was determined by evaluating activity over the range of temperatures spanning 25–90°C. Three replicate measurements for each of the experiments described above were made at each sampling interval, and replicate measurements did not vary by more than 5%.

### Crystallization and data collection

Crystals of EnoCa were obtained by the hanging drop vapor diffusion method at 18°C in 2 μl drops containing a 1:1 protein:reservoir solution ratio. The reservoir solution contained 0.8 ml of Bis-Tris propane buffer (0.1M, pH 9.0), 0.21M NaCl, and 28% PEG 1500. Crystals were cryoprotected by soaking them in the reservoir solution containing an additional 20% (v/v) glycerol, and they were then flash frozen in liquid nitrogen prior to data collection. The crystal composition was confirmed by SDS-PAGE and liquid chromatography–mass spectrometry analysis. Diffraction data were collected at 100 K at the Stanford Synchrotron Radiation Lightsource beamline 9-2, using the MARmosaic 325 CCD Detector (Menlo Park, CA). Data collected from EnoCa crystals were processed and scaled by XDS (Kabsch, 2010).

The structure was solved by molecular replacement (Winn et al., 2011) using the *Enterococcus hirae* enolase structure [PDB entry 1IYX (Hosaka et al., 2003)] as a search model. Model building was performed in Coot (Emsley et al., 2010). Coordinates were refined to reasonable stereochemistry at a resolution 2.30–3.04 Å using REFMAC5 (Murshudov et al., 1997). The structure was validated using MolProbity (Chen et al., 2010). All molecular images were calculated in PyMol (Delano, 2002). Calculation of root-mean-square deviations (r.m.s.d) was performed with the program LSQKAB (Winn et al., 2011). Structures are submitted to PDB entry 4YWS (native), 4Z17 (with PEP), 4Z1Y (with PGA).

### Amino acid sequence comparison and homology modeling

Amino acid sequences of enolases from *P. furiosus* (NP_577944), *T. maritima* (NP_228685), *T. aquaticus* (ZP_03497734), *Plasmodium falciparum* (XP_001347440), *Escherichia coli* (1E9I_A), *Candida albicans* (XP_711912), *S. cerevisiae* (1EBG_A), and *Trypanosoma brucei* (2PTW_A) were obtained from the NCBI/BLAST/BLASTP server.^1^ The ProtParam tool, available from the ExPASy server,^2^ was used to calculate the percentage amino acid composition (Gasteiger et al., 2003). Homology models containing one subunit of enolase from *P. furiosus*, *T. maritima*, *T. aquaticus*, *P. falciparum*, *C. albicans*, *S. cerevisiae*, and *T. brucei* were generated by SWISS-MODEL (Arnold et al., 2006).

### Structural rigidity analysis

Homology models were used to perform structural rigidity analysis. The program Floppy Inclusion Rigid Substructure Topography (FIRST) (Jacobs et al., 2001) was used to perform flexibility analysis and to calculate the number of probable (i) hydrogen bonds, (ii) rigid clusters, (iii) sites in the largest rigid cluster, and (iv) the total independent degrees of freedom. Using covalent bonds, hydrophobic tethers, hydrogen bonds, and salt bridges, FIRST defines the constraint network. Based on the constraint network, the program identifies compared parameters of rigid and flexible regions of the protein (Jacobs et al., 2001; Rader et al., 2002). Relationships between calculated and measured protein parameters and optimal growth temperature were determined using XL Stat (ver. 2008.7.03). Pearson correlation coefficients and *P*-values were generated from 1000 permutations of the data.

### Evolutionary analyses

Enolase-1 sequences were compiled from the DOE-IMG database using enolase-1 sequence from *E. coli* K12 (NP_417259) as a query. All representative sequences were aligned using ClustalX (ver. 2.0) (Larkin et al., 2007) employing the Gonnett substitution matrix with default parameters. A neighbor-joining tree was used to empirically identify sequences that represent the primary phylogenetic lineages. Representative enolase-1 sequences were realigned as described above and the alignment block was subjected to evolutionary model prediction using ProtTest (ver. 2.4).(Abascal et al., 2005) Phylogenetic reconstruction was performed with the neighbor-joining method specifying the JTT substitution matrix and gamma distributed rate variation (γ = 0.93) with MEGA4 (Tamura et al., 2007) The pairwise deletion option was specified and enolase-2 sequences from *Methanothermobacterium thermoautotrophicum* strain delta H and *Archaeoglobus fulgidus* DSM 4304 served as out groups. The phylogenetic tree was projected from 100 bootstrap replicates using FigTree (ver. 1.2.2).^3^

## Results

### Thermophilic origin and properties of thermal adaptation of enolase from *C. aurantiacus*

**Figure 1:**
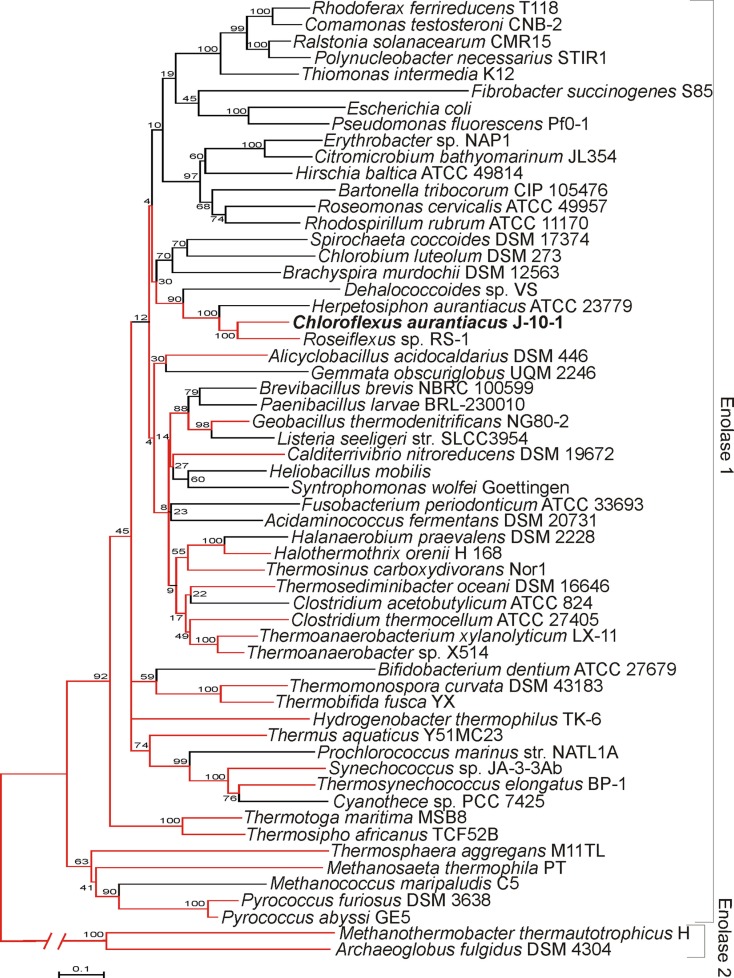
**Phylogenetic reconstruction of representative enolase-1 sequences as determined using the neighbor-joining method**. Lineages represented by thermophiles are colored red. Parsimony was used to designate thermophilic lineages in cases where both thermophilic and non-thermophilic taxa were present. Bootstrap support values are designated at nodes.

EnoCa shares significant sequence identity with enolases from other organisms distributed across Bacteria and Archaea (alignment not shown). Phylogenetic reconstruction of representative bacterial and archaeal enolase-1 sequences, when rooted with enolase-2, reveal a number of early branching lineages that are derived from thermophilic or hyperthermophilic organisms (Figure 1). Such an observation is consistent with a thermophilic origin for enolase-1. EnoCa-1 from *C. aurantiacus*, which is characterized here, forms a lineage with other green non-sulfur bacteria that branches late among thermophilic enolase. Nevertheless, these results suggest that the properties of EnoCa are likely to reflect those of the thermophilic ancestor to a greater extent than more recently derived mesophilic representatives.

The amino acid composition and flexibility analysis of EnoCa together with enolases from *P. furiosus*, *T. maritima*, *T. aquaticus*, *P. falciparum*, *C. albicans*, *S. cerevisiae*, and *T. brucei* are given in Table 1. Despite the high level of sequence conservation among enolases, EnoCa has a higher percentage of aliphatic amino acids when compared to enolases from non-thermophilic taxa. The Pearson correlations (*r*) between optimal growth temperature of microorganisms and parameters associated with EnoCa properties have been calculated to establish positive and negative relationships between these parameters. The results indicate that hydrophobocity indices, aliphatic indices, the total number of sites in the rigid cluster, and the total independent degrees of freedom varied positively and to a significant extent (*P* < 0.05) with the optimal growth temperature of the strains. In contrast, the relative abundance of polar amino acids, hydrogen bonds, and total number of clusters in the rigid cluster varied inversely with the optimal growth temperature of the strains (Table S3 in Supplementary Material). In addition, the EnoCa structure reveals differences relevant to thermostable proteins, such as the residues that form the loops (138–143, 189–207, and 247–268 in EnoCa) being shorter than the corresponding residues of *S. cerevisiae* enolase (Figure 3; Figure S6 in Supplementary Material).

**Table 1:** **Amino acid composition and flexibility analysis of enolases from different microorganisms**.

Organism	Growth temp, °C	pI	Amino acid composition, %							Flexibility analysis			
			Amino acids				Residues		AI^f^	H bonds	Rc	#Rc	df
			Hydrophobic^a^	Charged^b^	Polar^c^	Gly	Neg^d^	Pos^e^	
*P. furiosus*	100	4.98	48.8	27.4	14.7	9.1	64	45	97.74	295	1657	155	622
*T. maritima*	80	4.93	47.2	28	16.6	8.2	66	48	98.01	290	1575	526	656
*T. aquaticus*	70	5.01	47.5	27.7	13.7	11.1	64	47	98.27	296	1590	496	590
***C. aurantiacus***	**55**	**4.99**	**48.3**	**25.1**	**17.4**	**9.2**	**58**	**42**	**99.25**	**354**	**915**	**3405**	**525**
*E. coli*	37	5.32	45.6	26.4	17.1	10.9	59	48	89.12	344	1468	908	535
*C. difficile*	37	4.58	46.7	26	16.3	10.9	68	41	96.42	343	1389	1608	496
*P. falciparum*	37	6.21	45.8	25.1	21.7	7.4	55	53	97.35	361	905	3980	502
*C. albicans*	37	5.54	46.5	25.7	18.9	8.9	57	49	92.09	392	911	3620	455
*S. cerevisiae*	30	6.17	46	27.1	18.4	8.5	56	51	90.69	405	852	3826	406
*T. brucei*	27	5.93	43.8	26.6	20	9.6	56	51	84.41	399	589	4647	386

*^a^Total number of hydrophobic amino acids: alanine, leucine, isoleucine, valine, proline, phenylalanine, tyrosine, tryptophan, and methionine*.

*^b^Total number of charged amino acids: asparagine, glutamate, arginine, and lysine*.

*^c^Total number of polar amino acids: asparagine, glutamine, serine, threonine, and cysteine*.

*^d^Total number of negatively charged amino acids: asparagine and glutamate*.

*^e^Total number of positively charged amino acids: arginine and lysine*.

^f^AI – The aliphatic index of proteins is defined as the relative volume occupied by aliphatic side chains (alanine, valine, isoleucine, and leucine)

*pI, principle isoelectric point; neg, negative; pos, positive; AI, aliphatic index; H bonds, hydrogen bonds; Rc, rigid clusters; #Rc, total number of sites in rigid cluster; df, total independent degrees of freedom*.

*Bold font indicates that the data presented in the tables are from this work*.

### Biochemical characterization of enolase from *C. aurantiacus*

**Figure 2:**
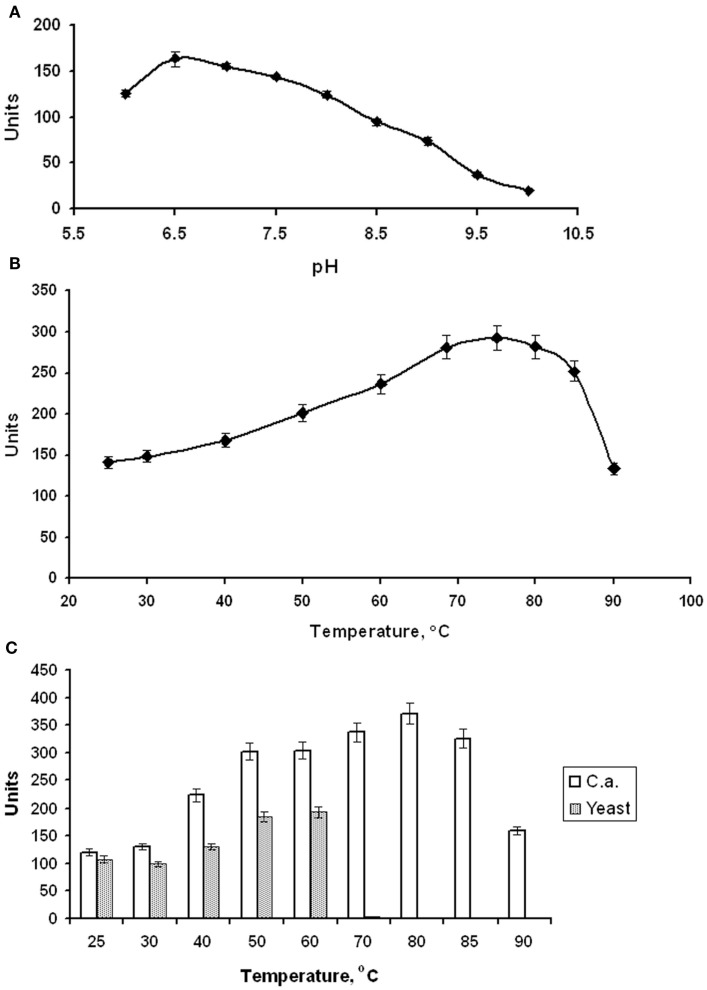
**Properties of *C. aurantiacus* enolase**. **(A)** Determination of pH optimum. **(B)** Thermostability of *C. aurantiacus* enolase. **(C)** Comparison of temperature optimum of *C. aurantiacus* and *Saccharomyces cerevisiae* enolases.

EnoCa was purified as a dimer with a molecular weight of ~92–96 kDa (Table 2; Figures S1–S3 in Supplementary Material). The subunit molecular weight of enolase was determined to be ~46.0 kDa using SDS-gel electrophoresis (Figure S2 in Supplementary Material). The activity of EnoCa was highest (124 ± 5 U mg^−1^ of protein at 25°C) in Bis-Tris propane buffer, with roughly a 25 and 47% decrease in activity when the enzyme was exchanged in Tris-HCl and HEPES, respectively. EnoCa activity was examined over a pH range of 6.0–10.0 in 50 mM Bis-Tris propane; the optimum pH for the catalytic activity of enolase was determined to be 6.5 (Figure 2A). EnoCa exhibited thermostability at temperatures up to 90°C, as indicated by retention of ~45% of the activity at this temperature when compared to that at 75°C. The temperature optimum for the assay reactions in 50 mM Bis-Tris propane buffer (pH 6.5) was 80°C in comparison to 55°C for the *S. cerevisiae* enolase (Figures 2B,C).

**Table 2:** **The purification of EnoCa**.

Stages of purification	Total protein, mg	Activity			Degree of purification
		Specific^a^, U/mg	Total, units	Yield, %	
Crude extract	570.1 ± 13.5	0.76 ± 0.03	433 ± 15	100	1
Chromatography on Q-sepharose	250.3 ± 9.8	1.28 ± 0.05	320 ± 12	74 ± 2	2 ± 0
Gel filtration on sephacryl S-300	20.8 ± 0.8	12.4 ± 0.3	258 ± 11	59 ± 2	16 ± 1
Chromatography on octyl-sepharose	1.1 ± 0.1	147 ± 6	162 ± 7	37 ± 1	213 ± 2

*^a^1 unit of enolase activity (μmol PEP min^−1^) was measured at 25°C*.

The *K*_m_ of EnoCa for 2-PGA and Mg^2+^ at 25 and 80°C were determined from measurements of initial rates of the reactions using the Lineweaver-Burk method (Lineweaver and Burk, 1934). EnoCa displayed classical Michaelis–Menten kinetics for both 2-PGA and Mg^2+^. The enzyme had a lower *K*_m_ for both 2-PGA (0.035 ± 0.00 mM) and Mg^2+^ (1.9 ± 0.3 mM) at 80°C, when compared to the *K*_m_ for these substrates as determined at 25°C (0.16 ± 0.01 and 2.5 ± 0.2 mM, respectively). As expected, the *V*_max_ for 2-PGA (50 ± 1 μmol min^−1^ mg^−1^) and Mg^2+^ (36 ± 2 μmol min^−1^ mg^−1^) was higher at 80°C, when compared to that at 25°C (9 ± 1 μmol min^−1^ mg^−1^ and 17 ± 2 μmol min^−1^ mg^−1^, respectively) (Table 3; Figures S4 and S5 in Supplementary Material). It should be noted that concentrations of at least 10 mM Mg^2+^ inactivated EnoCa (data not shown).

**Table 3:** **Properties of organisms from which enolase have been characterized, and properties of the purified enzymes**.

Organism	Growth temperature, °C	Specific activity, U/mg	MW, kDa		*K*_m_, 10^−3^M		pH_opt_	Temperature, °C	
			Subunit	Total	PGA	Mg^2+^		Opt	Stab
*P. furiosus*	100	14	45	90	0.4	n/a^a^	8.1	> 90	100
*T. maritima*	80	250	48	345^b^	0.07	0.03	7.5	90	94
*T. aquaticus*	70	450–900	44	352^b^	2.8^c^/3.5^d^	1.5^c^/0.9^d^	7.2^e^/8.5^f^	70	100
***C. aurantiacus***	**55**	**150–300**	**46**	**92**	**0.158^c^/0.035^d^**	**2.5^c^/1.9^d^**	**6.5**	**80**	**75**
*E. coli*	37	180	46	90	0.1	2.0	8.1	n/a	n/a
*C. difficile*	37	450	50	300^b^	3	2.0	7.6	55	70
*P. falciparum*^e^	37	30	50	100	0.041	0.18	7.4–7.6	n/a	n/a
*C. albicans*	37	35	46	100	0.38	0.286	6.8	n/a	n/a
*S. cerevisiae*	30	130	46	90	0.057	0.43	7.5	50	n/a
*T. brucei*	27	85	46	90	0.054	0.36	7.7	n/a	n/a

*^a^Not applicable*.

*^b^Total molecular weight for octameric structure*.

*^c^Measured at 25°C*.

*^d^Measured at optimum temperature*.

*^e^Expressed in *E. coli**.

*^f^Obtained at the optimum temperature*.

*MW, molecular weight; Opt, optimum; Stab, temperature stability*.

*Bold font indicates that the data presented in the tables are from this work*.

### Structural characterization of enolase from *C. aurantiacus*

**Figure 3:**
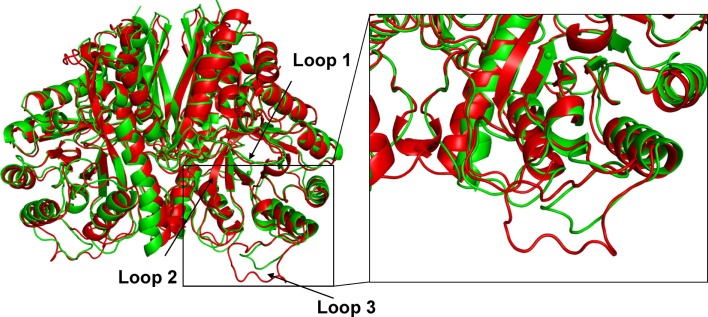
**Comparison of crystal structure of *C. aurantiacus* and *Saccharomyces cerevisiae* enolase**. Difference in the secondary-structure elements of *C. aurantiacus* enolase (in green) and *S. cerevisiae* enolase (1EBH; in red). The two structures are superimposed and the difference in the loop with residues 189–207 and L3 loop (residues 247–268) are shown. Insert shows close-up view of the loop L3.

EnoCa was crystallized under multiple conditions; however, the best crystals were obtained using 0.21M NaCl and 28% PEG 1500. High-resolution crystal structures were obtained for the apo protein as well as proteins with 2-PGA and PEP bound in the active site. The enolase crystals belonged to space group *I*4, which contained two monomers per asymmetric unit assembled into one homodimer (Table S4 in Supplementary Material). Each monomer of EnoCa contains an amino terminal domain that consists of a three-stranded β-sheet packed against three α-helices and a carboxy terminal domain that consists of an eightfold α/β-barrel (Figure 3); both domain features are typical of the enolase superfamily.

**Figure 4:**
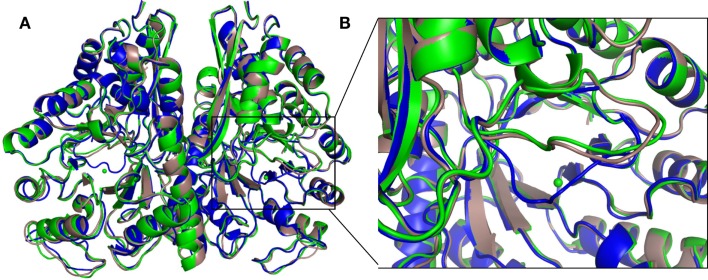
**Differences in the overall crystal structures of *C. aurantiacus* enolase**. Superposition of apo (green), 2-PGA (gray), and PEP (blue) structures **(A)**. Close-up view of the L1 loop. In the PEP structure, the L1 loop is shown in close conformation **(B)**.

The structure of the apo protein and that containing 2-PGA (2-PGA EnoCa structure) of EnoCa exhibits the so-called “open” conformation, where the L1 loop is away from the active site. The EnoCa structure containing PEP (PEP EnoCa structure) exhibits the so-called “closed” conformation, where the L1 loop is located closer to the active site (Figures 4A,B). Upon superposition with the apo EnoCa structure in the open conformation, the L1 loop in the 2-PGA EnoCa structure (open conformation) displays an r.m.s.d. of 0.38 Å, whereas the PEP EnoCa structure superimposed on the apo EnoCa structure (closed conformation) has r.m.s.d. 3.1 Å.

**Figure 5:**
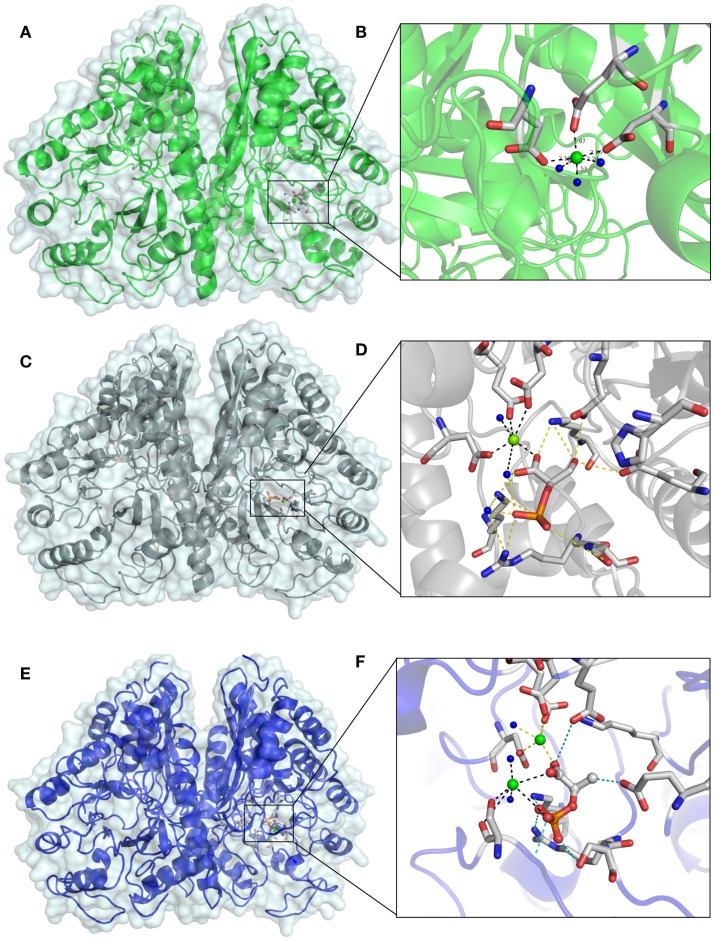
**Differences in the active site of crystal structures of *C. aurantiacus* enolase**. The active sites of native EnoA in green **(A,B)** 2-PGA structure in gray **(C,D)**, and PEP structure in blue **(E,F)** are shown in overall **(A,C,E)** and close view **(B,D,F)**.

The remaining electron density was visible in the active site, after refinement of all protein structures. In the beginning of the apo EnoCa structure refinement, the difference map peaks were refined placing Mg^2+^(I) atom in coordination with Asp241, Glu285, and Asp313, and then three water molecules in an octahedral manner (Figures 5A,B). The nature of the metal ion could not be unambiguously determined from the metal–oxygen distances of between 2.4 and 2.5 Å (Dokmanic et al., 2008). Hence, the octahedral coordination sphere, the presence of Mg^2+^ in the crystallization buffer, and the fact that Mg^2+^ is the natural ligand of enolase, is consistent with the modeling of the difference density peak as an Mg^2+^ ion (Brewer, 1981; Wedekind et al., 1995; Larsen et al., 1996). Like the apo EnoCa structure, a metal ion was mapped to the active site in the 2-PGA and PEP structures. However, for the same reasons described above for the apo EnoCa structure, unambiguous determination of the metal ion was not possible in the 2-PGA and PEP EnoCa structures.

The presence of 2-PGA and PEP in the active site of EnoCa was confirmed by calculation of a simulated-annealing OMIT map. The simulated-annealing OMIT map shows electron density that correlates with the presence of 2-PGA and PEP molecules (data not shown). As was mentioned before, the L1 loop in 2-PGA EnoCa structure was refined in the open conformation. 2-PGA in the active site is coordinated with Glu165, Glu206, Lys338, Arg367, and Ser368. Additionally, 2-PGA interacts with water molecules, and the carboxyl molecule involved in coordination of conformational Mg^2+^(I) (Figures 5C,D).

In contrast to the 2-PGA EnoCa structure, PEP was coordinated with the second catalytic metal atom in the active site. The second atom was identified as Mg^2+^. Mg^2+^(II) was coordinated with Ser43, water molecules, and phosphate atoms from PEP supporting the octahedral coordination sphere. PEP itself is bound by Glu165, Glu206, Lys338, Arg367, and Ser368 (Figures 5E,F). It should be noted that the electron density was absent for the Ser43 in subunit A of the PEP EnoCa structure due to instability of the L1 region. Nevertheless, the Ser43 was modeled in subunit A with reduced occupancy (0.5).

## Discussion

Evolutionary analysis of archaeal and bacterial enolase-1 protein representatives, when rooted with representative enolase-2 proteins, indicate that the earliest branching lineages derive from thermophilic taxa indicating that the enzyme likely has a thermophilic origin. Enolase-1 from the thermophile *C. aurantiacus*, as characterized here, branches late among the thermophilic ancestry of the enzyme, but prior to when the widespread diversification of organisms harboring this enzyme into lower temperature environments. Thus, it is likely that enolase-1 isolated from *C. aurantiacus* harbors biochemical and structural properties that are more reflective of the thermophilic ancestor than mesophilic enzymes.

Despite the clear indication that EnoCa is derived from a thermophilic ancestor, the molecular properties of the enzyme exhibit a number of similarities with characterized enzymes from mesophiles. For example, the subunit molecular weights are similar in enolase from *E. coli* (Wold and Ballou, 1957a), *P. furiousus* (Peak et al., 1994), *S. cerevisiae* (Chin et al., 1981), *T. brucei* (Hannaert et al., 2000), and *P. falciparum* (Pal-Bhowmick et al., 2004), all of which vary from 45.0 to 46.4 kDa. Moreover, the pH optima of enolases from various organisms are also similar among enzymes (Wold, 1971; Kustrzeba-Wojcicka and Golczak, 2000) and tend to fall within the ~6 to 7 range with activity decreasing sharply in more acidic medium and less so in more alkaline solutions. Since enolase is a cytoplasmic enzyme, this finding likely reflects similar cytoplasmic pH in these organisms and may indicate similar cytoplasmic pH since early in life history. The *K*_m_ value for 2-PGA as a substrate for EnoCa is 0.16 mM at 25°C and 0.03 mM at 80°C, which compares well with the *K*_m_ value for *S. cerevisiae* enolase (0.12 mM) at 25°C (Wold and Ballou, 1957b). Enolase from *T. aquaticus* and *C. difficile* have *K*_m_ values for 2-PGA of 2.8 and 3.0 mM respectively (Stellwag et al., 1973; Green et al., 1993), which is one order of magnitude higher than that observed for *C. aurantiacus* and *S. cerevisiae*.

Bivalent metal ions have been shown to be necessary for the activation of enolase. Specifically, Mg^2+^ has shown to be the strongest activator for all known enolases (Wold and Ballou, 1957b; Faller et al., 1977). Other bivalent metal ions activated homologous enolase enzymes to varying extents (Faller et al., 1977; Brewer, 1981). *K*_m_ values for the MgCl_2_ for EnoCa are 2.5 (at 25°C) and 1.5 mM (at 80°C), similar to those from *E. coli* and *C. difficile* which are 2.0 mM (at 25°C) but an order magnitude higher than for mesophilic bacteria (Table 3). The decrease in the *K*_m_ values for 2-PGA and Mg^2+^ with an increase of the temperature approaching the temperature optimum of the catalytic reaction has been shown for enolase from *T. quaticus* (Stellwag et al., 1973) and *T. maritima* (Schurig et al., 1995). Together, these observations indicate adaptation of the thermostable enzymes with respect to their structural properties and activity, showing maximum catalytic efficiency and stability at high temperatures. Similar to the effect of Mg^2+^ on activity of *S. cerevisiae* enolase (Faller et al., 1977; Vinarov and Nowak, 1998), the increase in the Mg^2+^ concentration (higher than 10 mM) inhibited the activity of EnoCa. The inhibitory metal binding site has been identified in structural studies conducted on enolase from *T. brucei* (Giotto et al., 2003).

Enolase from the thermophile *C. aurantiacus* (growth optimum = 55°C) exhibits thermostability and a higher temperature optimum (enolase *T*_opt_ = 80°C) than enolase from the thermophile *T. aquaticus* (growth optimum = 70°C; enolase *T*_opt_ = 70°C), but a lower temperature optimum than enolase from the hyperthermophiles *P. furiosus* (growth optimum = 100°C; enolase *T*_opt_ = >90°C) and *T. maritima* (growth optimum = 80; enolase *T*_opt_ = 90°C) (Table 3). There are several differences in the properties of enolase proteins observed in the primary sequence and structure that may account for the differences in the observed thermostability. For example, the optimal growth temperature of strains significantly correlates with the hydrophobicity index (e.g., number of hydrophobic residues in the protein sequence), aliphatic index (Ikai, 1980), number of rigid clusters in inferred structures, and total independent degree of freedom. These differences would support enhanced thermal stability, since bulkier hydrophobic amino acid side-chains can support stronger hydrophobic interactions in the protein interior (Jaenicke, 1991; Vieille and Zeikus, 2001). Similar adaptations at the structural level have been observed in ribulose-1,5-bisphosphate carboxylase/oxygenase in response to temperature (Miller, 2003). The conformational entropy of the protein molecule increases with elevated temperatures, and this can be attributed to the high degree of disorder observed in the protein–solvent interactions. Therefore, the increase in conformational entropy may have deleterious effects on the structure of the protein molecule by altering its active configuration and leading to its denaturation at elevated temperatures. Hence, to maintain the structural integrity, the thermophilic protein needs to adopt certain strategies that will act as a control mechanism to lower the conformational entropy and prevent it from denaturing at higher temperatures (Kumar and Nussinov, 2001). Increased rigidity of the thermophilic proteins can be considered the first step toward the reduction of the conformational disorder of the protein molecule. This can be achieved by stronger interactions within the protein interior. The rigid clusters, calculated by the FIRST software, are formed from a collection of atoms connected by non-rotatable bonds varying in size from hundreds and thousands of atoms forming a rigid protein core, down to a single atom (Jacobs et al., 2001). Thus, a high temperature stability is the result of adaptation at the structural level in the response to high temperature (Ikai, 1980; Kumar and Nussinov, 2001; Vieille and Zeikus, 2001; Miller, 2003).

In conclusion, the results from the structural analysis of *C. aurantiacus* enolase presented here are consistent with its thermophilic ancestry. For example, the *C. aurantiacus* enolase has a decreased number of amino acids that comprise flexible loops (Figure 3). Among proteins that are isolated from thermophiles, it is common to observe an overall greater proportion of amino acids involved in well-defined secondary structure (Ikai, 1980; Vieille and Zeikus, 2001). This is manifested not only in the lack of extensive solvent-exposed loop regions, but also in a greater extent of secondary structure. Increase in the helical content along with shortening of loop regions results in a decrease in conformational entropy. The other feature that was identified in the structural analysis is the absence of second, catalytic Mg^2+^(II) atom at the active site in the structures containing 2-PGA. It has been reported that the *S. cerevisiae* and *E. histolytica* enolase structures contain the 2-PGA that was not coordinated with Mg^2+^(II) in the active site. This fact might be explained by the pre-catalytic step of the reaction sequence prior to binding of metal II and closure of L1.

## Conflict of Interest Statement

The authors declare that the research was conducted in the absence of any commercial or financial relationships that could be construed as a potential conflict of interest.

## Supplementary Material

The Supplementary Material for this article can be found online at http://journal.frontiersin.org/article/10.3389/fbioe.2015.00074/abstract

## Footnotes

^1^http://blast.ncbi.nlm.nih.gov

^2^http://web.expasy.org/protparam/

^3^http://tree.bio.ed.ac.uk/software/figtree/

## Abbreviations

EnoCa, enolase-1 from *Chloroflexus aurantiacus*; FIRST, floppy inclusion rigid substructure topography; 2-PGA, 2-phosphoglycerate; PEP, phosphoenolpyruvate; PAGE, polyacrylamide gel electrophoresis; PEG, polyethylene glycol; PDB, protein data bank; R.m.s.d.s, root-mean-square deviations; SDS-PAGE, sodium dodecyl sulfate polyacrylamide gel electrophoresis.
