# Supplementary material for: Biochemical and Structural Characterization of Enolase from Chloroflexus aurantiacus: Evidence for a Thermophilic Origin
Source: Front Bioeng Biotechnol. 2015 Jun 1;3:74. doi: 10.3389/fbioe.2015.00074 (PMC4450660; doi:10.3389/fbioe.2015.00074)

**Supporting information**

Biochemical and Structural Characterization of Enolase from *Chloroflexus aurantiacus:* Evidence for a Thermophilic Origin

Oleg A. Zadvornyy^1,4^, Eric S. Boyd^2^, Matthew C. Posewitz^3^, Nikolay A. Zorin^4^ and John W. Peters^1^*

Table S1. Modified Castenholz Medium for C. aurantiacus J.10.fl cultivation.

| Medium compounds | g/L |
| --- | --- |
| MgSO_4_ x 7H_2_O | 0.1 |
| CaCl_2_ | 0.04 |
| KCl | 0.125 |
| NaCl | 0.25 |
| KH_2_PO_4_ | 0.08 |
| (NH_4_)_2_SO_4_ | 0.125 |
| Glycyl-glycin | 1.0 |
| Yeast extract | 2.0 |
| Vitamin B_12_ | 2 μg |
| Trace element solution SL 8 | 1 ml |

Table S2. Trace element solution *C*. aurantiacus J.10.fl cultivation.

| Medium compounds |  |
| --- | --- |
| Na-EDTA x 2H_2_O | 5 g |
| ZnCl_2_ | 67 mg |
| FeCl_3_ x 4 H_2_O | 1.5 g |
| MnCl_2_ x 4 H_2_O | 99 mg |
| H_3_BO_3_ | 62 mg |
| CuCl_2_ x 2 H_2_O | 17 mg |
| Na_2_MoO_4_ x 2 H_2_O | 36.3 mg |
| CoCl_2_ x 6 H_2_O | 120 mg |
| Distilled water | 1 L |

**Table S3.** Pearson correlations (*r*) between parameters associated with purified enolase proteins with those relationships that are significant (*P*<0.05) highlighted in bold. The data used in this analysis is from Table 3.

| Variables^a^ | Temp | pI | Hid | Charged | polar | Gly | neg | pos | AI | H bonds | Rc | #Rc | df |
| --- | --- | --- | --- | --- | --- | --- | --- | --- | --- | --- | --- | --- | --- |
| Temp | **1.000** | -0.578 | **0.797** | 0.563 | **-0.703** | -0.039 | 0.604 | -0.362 | **0.652** | **-0.871** | **0.739** | **-0.751** | **0.882** |
| pI | -0.578 | **1.000** | **-0.655** | -0.241 | **0.794** | -0.549 | **-0.874** | **0.888** | -0.546 | **0.715** | **-0.731** | **0.745** | **-0.664** |
| HI | **0.797** | **-0.655** | **1.000** | 0.162 | **-0.678** | 0.006 | 0.524 | **-0.644** | **0.849** | **-0.668** | 0.603 | -0.585 | **0.720** |
| Charged | 0.563 | -0.241 | 0.162 | **1.000** | -0.621 | 0.174 | 0.517 | 0.065 | 0.006 | -0.535 | 0.579 | -0.623 | 0.460 |
| polar | **-0.703** | **0.794** | **-0.678** | -0.621 | **1.000** | -0.608 | **-0.781** | **0.641** | -0.443 | **0.751** | **-0.827** | **0.856** | **-0.662** |
| Gly | -0.039 | -0.549 | 0.006 | 0.174 | -0.608 | **1.000** | 0.444 | -0.521 | -0.144 | -0.219 | 0.399 | -0.439 | 0.055 |
| neg | 0.604 | **-0.874** | 0.524 | 0.517 | **-0.781** | 0.444 | **1.000** | **-0.665** | 0.516 | **-0.782** | **0.827** | **-0.821** | **0.684** |
| pos | -0.362 | **0.888** | **-0.644** | 0.065 | **0.641** | -0.521 | **-0.665** | **1.000** | -0.494 | 0.423 | -0.448 | 0.459 | -0.372 |
| AI | **0.652** | -0.546 | **0.849** | 0.006 | -0.443 | -0.144 | 0.516 | -0.494 | **1.000** | **-0.687** | 0.528 | -0.470 | **0.724** |
| H bonds | **-0.871** | **0.715** | **-0.668** | -0.535 | **0.751** | -0.219 | **-0.782** | 0.423 | **-0.687** | **1.000** | **-0.909** | **0.906** | **-0.967** |
| Rc | **0.739** | **-0.731** | 0.603 | 0.579 | **-0.827** | 0.399 | **0.827** | -0.448 | 0.528 | **-0.909** | **1.000** | **-0.993** | **0.871** |
| # Rc | **-0.751** | **0.745** | -0.585 | -0.623 | **0.856** | -0.439 | **-0.821** | 0.459 | -0.470 | **0.906** | **-0.993** | **1.000** | **-0.864** |
| df | **0.882** | **-0.664** | **0.720** | 0.460 | **-0.662** | 0.055 | **0.684** | -0.372 | **0.724** | **-0.967** | **0.871** | **-0.864** | **1.000** |

^a^ Abbreviations: Temp - Optimum Growth Temperature, pI - principle isoelectric point, Hid - total number of hydrophobic amino acids, AI - Aliphatic Index, H bonds - hydrogen bonds, Rc - Rigid clusters, # Rc - total number of sites in rigid cluster, df - total independent degrees of freedom.

Table S4. Data collection and refinement statistics*.

| Data Collection | Apo  4YWS | PEP  4Z17 | PGA  4Z1Y |
| --- | --- | --- | --- |
| Wavelength (Å) | 0.97946 | 0.95369 | 0.95369 |
| Unit cell parameters (angstrom, degree, : | *a* = *b*= 146.32  c= 102.99  α= β= γ= 90 | *a* = *b*= 146.28  c= 101.78  α= β= γ= 90 | *a* = *b*= 146.32  c= 101.88  α= β= γ= 90 |
| Space group | *I*4 | *I*4 | *I*4 |
| Resolution range (Å) | 37.76-2.45 | 37.69-2.65 | 37.75-2.53 |
| Total reflections | 221189 | 135547 | 167049 |
| Unique reflections | 39821 | 31118 | 35899 |
| R-merge(%) | 0.046 (0.433) | 0.073 (0.561) | 0.054 (0.473) |
| I/σ (I) | 27.9 (4.4) | 19.6 (3.2) | 21.8 (3.6) |
| Completeness (%) | 99.7 (98.2) | 99.6 (99.4) | 99.8 (99.0) |
| Redundancy | 5.6 (5.5) | 4.4 (4.3) | 4.7 (4.6) |
| *Refinement* | | | |
| Resolution limits (Å) | 37.76-2.45 | 37.69-2.65 | 37.7-2.53 |
| No of used reflections | 39821 | 31115 | 35899 |
| No of protein atoms | 6068 | 6421 | 6466 |
| R factor (%) | 19.9 | 19.3 | 21.4 |
| R free (%) | 24.0 | 23.8 | 25.2 |
| R.m.s. deviations from ideal values, bond lengths of refined atoms | 0.021 | 0.020 | 0.021 |
| Bond distance (Å) | 0.02 | 0.02 | 0.01 |
| Angels (Å) | 2.06 | 2.01 | 1.74 |

* Values in parentheses are for the highest resolution shell.

Figure S1. Purification of EnoCa. SDS-PAGE gel of the crude cell extract (1), supernatant of the cell free extract (2), and pellet after centrifugation of sonicated cells (3), and protein markers (4)

| A 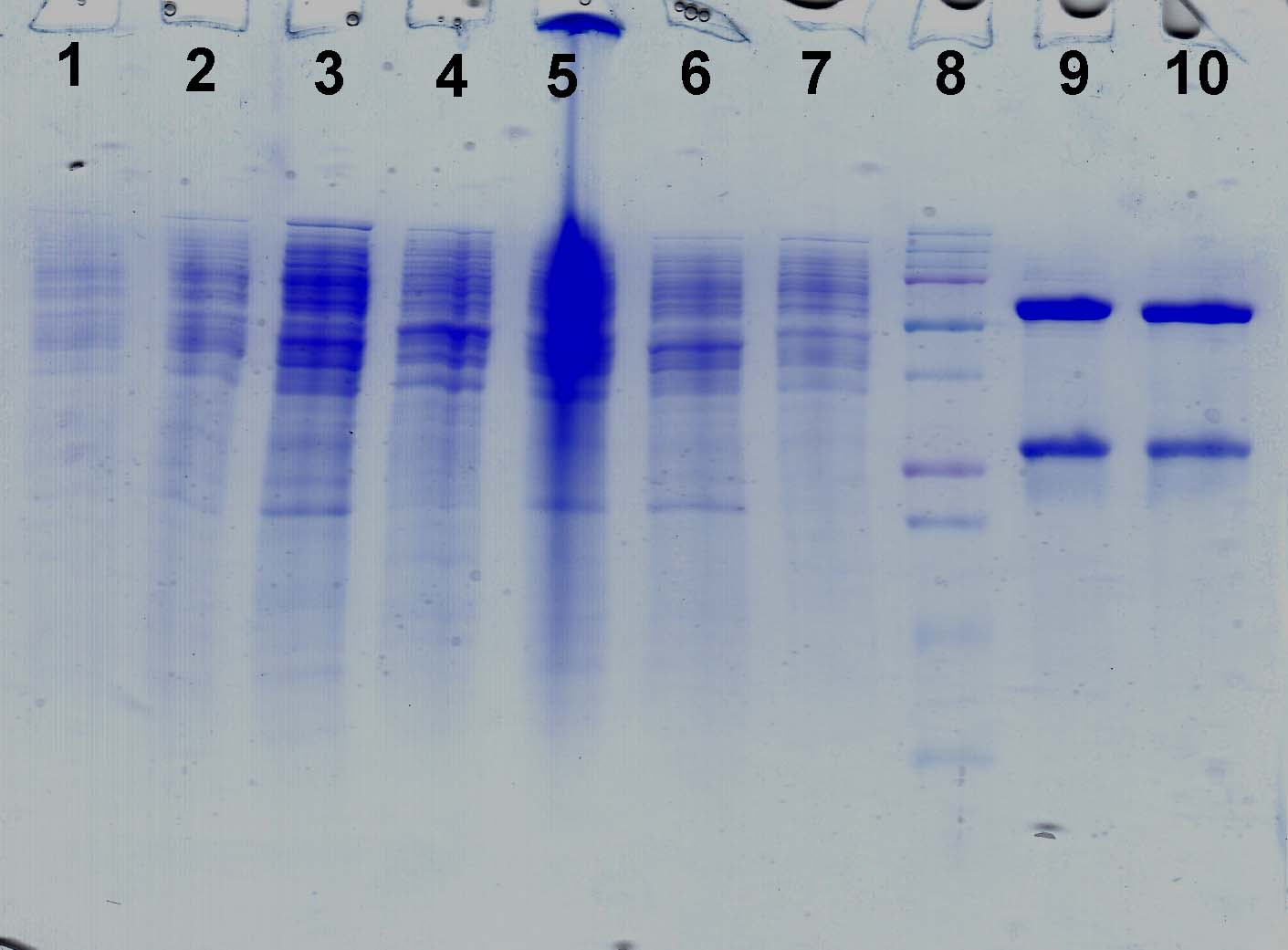 **1 234**  **50**  **37**  **kDa** |
| --- |

Figure S2. Purification of EnoCa. Native PAGE gel showing that the EnoCa is purified as a dimer at ~92 kDa. SDS-PAGE indicates that the EnoCa subunit is ~46 kDa.

| 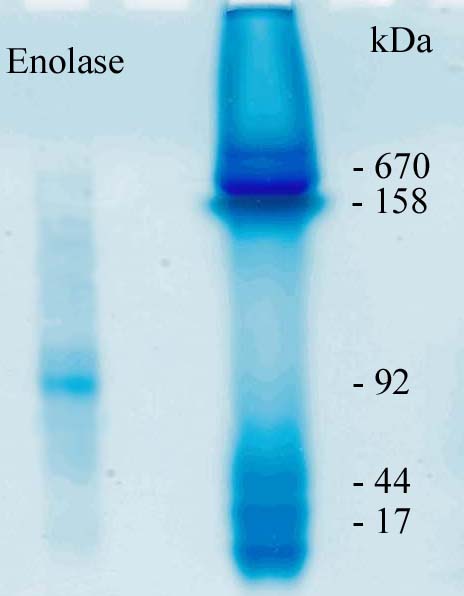 A | 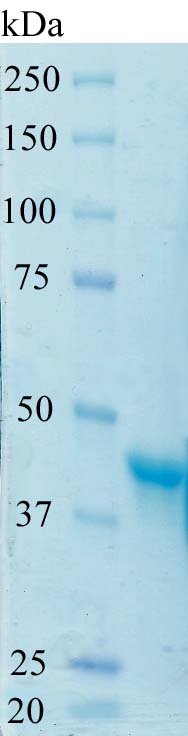B |
| --- | --- |

Figure S3. Purification of EnoCa on a Sephacryl S-300 gel filtration column. The protein standards are shown in blue, the EnoCa sample corresponded to ~100 kDa is shown in purple.

Figure S4. Effect of the temperature on the K_m_ values for 2-PGA. Determination of the K_m_ and V_max_ for 2-PGA at 25 °C (A) and 80 °C (B) were done using the Lineweaver-Burke method.

|  |
| --- |
| B |

Figure S5. Effect of temperature on the K_m_ values for Mg^2+^. Determination of the K_m_ and V_max_ for 2-Mg^2+^ at 25 °C (A) and 80 °C (B) were done using the Lineweaver-Burke method.

| A |
| --- |
| B |

Figure S6. Sequence alignment of enolases from *C. aurantiacus,* *P. furiosus*, *T. aquaticus*, *S. cerevisiae* and *E. hirae*. Significant loop regions in enolase structures are shown: 1 corresponds to the L1 loop of a cap domain; 2 and 4 correspond to the loops with different length; 3 and 5 correspond to the L2 and L3 loops, respectively.


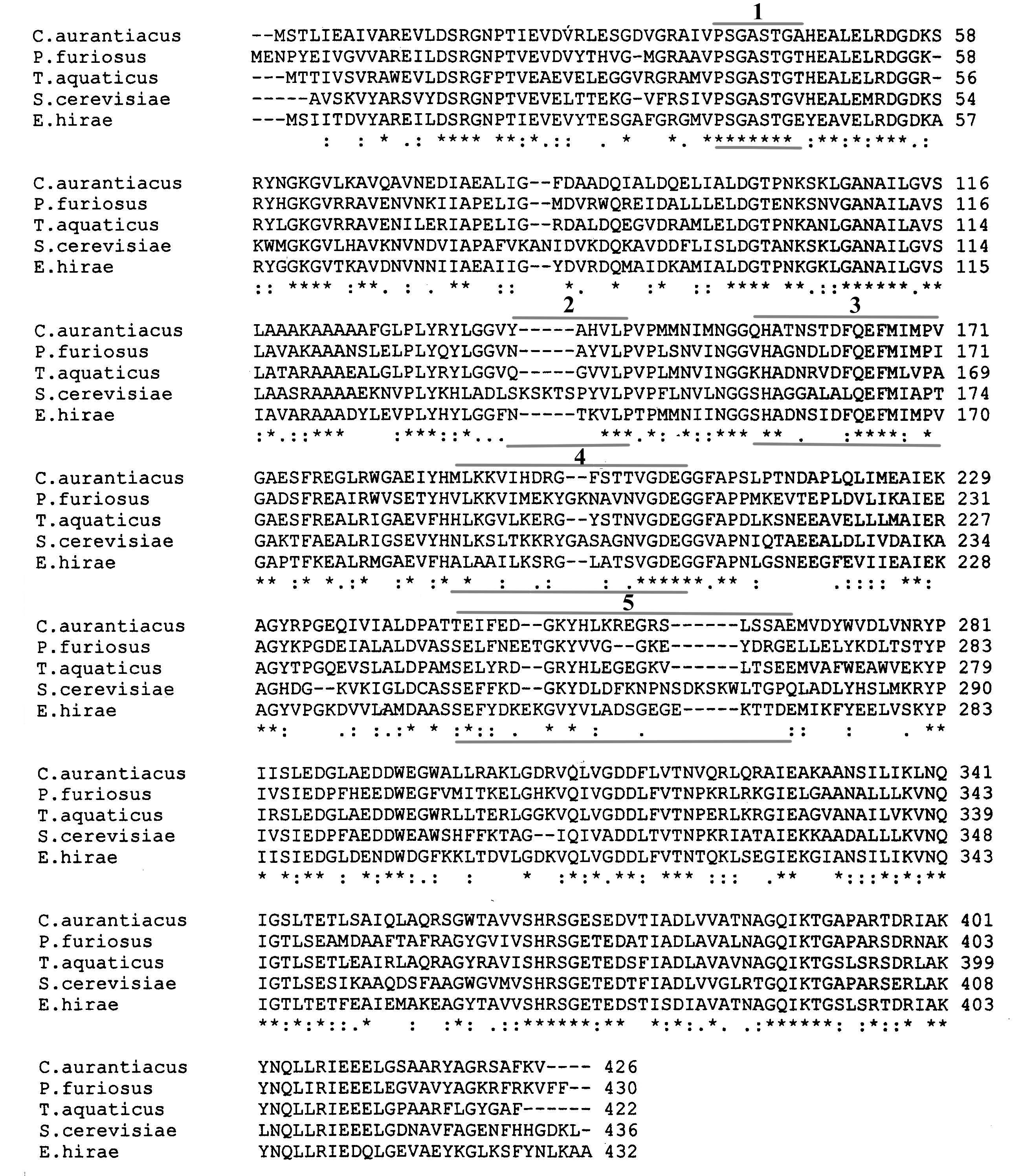

Supplement: Supplementary file 1 [file Data_Sheet_1.DOC]
